# Real-World Data on the Impact of COVID-19 on Endoscopic Procedural Delays

**DOI:** 10.14309/ctg.0000000000000365

**Published:** 2021-06-01

**Authors:** Rachel B. Issaka, Lauren D. Feld, Jason Kao, Erin Hegarty, Brandon Snailer, Gorav Kalra, Yutaka Tomizawa, Lisa Strate

**Affiliations:** 1 Clinical Research Division, Fred Hutchinson Cancer Research Center, Seattle, Washington, USA;; 2 Division of Gastroenterology, University of Washington School of Medicine, Seattle, Washington, USA;; 3 Seattle Cancer Care Alliance, Seattle, Washington, USA;; 4 University of Washington School of Medicine, Seattle, Washington, USA.

## Abstract

The initial surge of the coronavirus disease 2019 (COVID-19) pandemic prompted national recommendations to delay nonurgent endoscopic procedures. The objective of this study was to provide real-world data on the impact of COVID-19 on endoscopic procedures in a safety-net healthcare system and cancer center affiliated with a tertiary academic center.

**METHODS:**

This retrospective cohort study used a combination of electronic health record data and a prospective data tool created to track endoscopy procedures throughout COVID-19 to describe patient and procedural characteristics of endoscopic procedures delayed during the initial COVID-19 surge.

**RESULTS:**

Of the 480 patients identified, the median age was 57 years (interquartile range 46–66), 55% (n = 262) were male, and 59% self-identified as white. Colonoscopy was the most common type of delayed procedure (49%), followed by combined esophagogastroduodenoscopy (EGD) and colonoscopy (22%), and EGD alone (20%). Colorectal cancer screening was the most common indication for delayed colonoscopy (35%), and evaluation of suspected bleeding (30%) was the most common indication for delayed combined EGD and colonoscopy. To date, 46% (223/480) of delayed cases have been completed with 12 colorectal, pancreatic, and stomach cancers diagnosed. Sociodemographic factors, procedure type, and sedation type were not significantly associated with endoscopy completion. The median time to endoscopy after delayed procedure was 88 days (interquartile range 63–119) with no differences by procedure type.

**DISCUSSION:**

To minimize potential losses to follow-up, delayed, or missed diagnoses and to reduce progression of gastrointestinal diseases, all efforts should be used to ensure follow-up in those whose endoscopic procedures were delayed because of COVID-19.

## INTRODUCTION

During the initial surge of the coronavirus disease 2019 (COVID-19) pandemic, healthcare systems in the United States (US) rapidly adjusted to reduce disease transmission and reserve capacity for those infected (1). The US Surgeon General advised that all nonurgent medical procedures and surgeries be delayed (2), and a joint statement by the 4 US gastroenterology societies recommended rescheduling elective endoscopic procedures (3). Although initially delaying elective and nonurgent procedures was necessary, ongoing delays or cancellations could lead to other public health crises from preventable and chronic diseases (4,5).

Although it is challenging to measure the exact impact of COVID-19 on preventable and chronic diseases, COVID-19 has significantly influenced gastrointestinal (GI) disease management. Healthcare technology platform and modeling data indicated an initial 90% decline in colonoscopies and biopsies compared with a year earlier (6), an estimated 1.7 million missed colonoscopies, and an additional 4,500 deaths that could result from colorectal cancer alone over the next decade because of COVID-19-associated delays (7). System-level data on the impact of COVID-19 on endoscopic delays in the United States are needed to determine the real-world impact of these delays.

Understanding the real-world impact of COVID-19 on procedural delays is important because it can inform local policy, workforce assignments, and resource allocation (e.g., COVID-19 testing) (8). In addition, because some GI diseases disproportionately affect racial/ethnic minorities and lower socioeconomic individuals, system-level data may encourage implementation of creative solutions to ensure equitable access to care throughout the pandemic (9).

The primary aim of this study was to describe the impact of COVID-19 on endoscopic procedural delays in a safety-net healthcare system and cancer center both affiliated with an urban, tertiary academic institution. Specifically, we report the types of endoscopic procedures that were delayed at the peak of the COVID-19 pandemic, procedure indications, and the patient populations impacted by these delays. The secondary aim was to identify factors associated with endoscopy completion among patients who returned to care after initial COVID-19-related delays, time (days) to procedure completion, and complications from delays.

## METHODS

### Study design

We performed a retrospective cohort study of all endoscopic procedures delayed between March 18, 2020, and May 18, 2020, in accordance with national and local guidance for management of nonurgent procedures and surgeries during the initial COVID-19 surge. As per guidance from the gastroenterology associations, procedures for GI bleeding, dysphagia, cholangitis, and other urgent/emergent indications (n = 268 procedures) were completed during the study period and are not included in this report (see Supplemental Table 1, Supplementary Digital Content 1, http://links.lww.com/CTG/A625) (10). Data were abstracted from patient electronic health records (EHRs) and a home-grown database created to collect data prospectively throughout the COVID-19 pandemic. Institutional Review Board approval was obtained for a minimal-risk study according to institutional regulations, and individual informed consent was not required.

### Study setting and population

The study took place within Harborview Medical Center (HMC) and the Seattle Cancer Care Alliance (SCCA). HMC is a safety-net county teaching hospital system in Seattle, WA, with 7 primary care clinics that provide care to the most vulnerable residents in King County, including those who are lower-income, uninsured, and whose primary language is not English. SCCA is a member of the National Comprehensive Cancer Network, providing cancer prevention, treatment, and surveillance to individuals in the Pacific Northwest and beyond. Both HMC and SCCA are integrated with a single EHR through their affiliation with the University of Washington. All patients with delayed endoscopic procedures between March 18 and May 18 were included, and those with procedures delayed outside of this time window were excluded. Patients who completed urgent or emergent endoscopic procedures during this period were also excluded.

### Study covariates

The final data set included the date of the initially planned endoscopic procedure, procedure type, indication, date procedure completed, sedation type, pathology results, and demographic data including self-reported sex, race, ethnicity, and primary language. When applicable, our data set also included the date of emergency department use, indication for emergency care, hospitalization date, and indication for hospitalization. From the EHR and the home-grown database, we extracted patient demographic factors, procedure type (colonoscopy, esophagogastroduodenoscopy [EGD], combined EGD and colonoscopy, or other [flexible sigmoidoscopy, endoscopic ultrasound, endoscopic retrograde cholangiopancreatography, single-balloon enteroscopy, double-balloon enteroscopy, or video capsule endoscopy]), procedure indication, sedation type (anesthesia, moderate, or none), pathology results, emergency care, and hospitalization details.

### Statistical analysis

Patient demographic information was described as proportions or medians and interquartile ranges (IQRs). Days from procedure delay to completion, use of emergency care, or hospitalization was described using medians and IQR. Differences between groups were assessed using χ^2^, Student *t* test, and nonparametric median 2-sample tests as appropriate. We performed linear regression analysis to determine the relationship between time to endoscopy completion and procedure type and logistic regression analysis to determine the factors associated with completing an endoscopic procedure after COVID-19-related delays. Our multivariable analysis adjusted for age, sex, race, ethnicity, primary language, procedure type, and sedation type. Accompanying odds ratios (ORs), 95% confidence interval (CI), and *P* values were reported in all instances, and *P* values < 0.05 were considered statistically significant. We used Stata/SE (version 16.0; StataCorp LP, College Station, TX) statistical software for all analyses.

## RESULTS

Based on inclusion and exclusion criteria, 480 patients were eligible for this study. The median age was 57 years (IQR 46–66), 55% (n = 262) were male, 59% self-identified as white, 15% black, 10% Asian/Pacific Islander, 3% American Indian/Alaskan Native, 2% Other, and race was not reported by 11%. Within the cohort, 79% self-identified as non-Hispanic, 11% as Hispanic, and 10% did not identify their ethnicity; 84% were primary English speakers and 16% were nonprimary English speakers (see Supplemental Table 2, Supplementary Digital Content 1, http://links.lww.com/CTG/A625).

Colonoscopies were the most common type of delayed procedure (49%), followed by combined EGD and colonoscopy (22%), and EGD alone (20%) (Table 1). The most common procedural indications among delayed colonoscopies were colorectal cancer screening (35%), polyp surveillance (24%), and evaluation of inflammatory bowel disease (9%). For delayed EGD, the most common procedural indications were symptoms without warning signs (nausea/vomiting, abdominal pain, dyspepsia, and gastroesophageal reflux disease) (23%) and esophageal variceal screening or surveillance (22%). For delayed combined EGD and colonoscopy, the most common procedural indications were evaluation of suspected bleeding (e.g., iron deficiency anemia) (30%) and miscellaneous reasons (abnormal imaging, variceal or colorectal cancer screening, and surveillance) (20%). Among other delayed procedures, 48% (n = 21/44) were endoscopic retrograde cholangiopancreatography and EUS, the majority were to evaluate abnormal imaging findings (67%). Of all 480 delayed procedures, 53% (n = 252) were scheduled with moderate sedation, 46% (n = 223) with anesthesia, and 1% (n = 5) without sedation.

**Table 1.** Frequency and indications of delayed coronavirus disease 2019 procedures

	Total (n = 480)	
	N	%
Colonoscopy	234	49
CRC screening	83	35
History of colonic polyps	55	24
Inflammatory bowel diseases (Crohn's and ulcerative colitis)	22	9
Bleeding (BRBPR, IDA)	22	9
Abnormal FIT result	14	6
LGI symptoms (changes in stool, abdominal pain, chronic diarrhea, and weight loss)	11	5
CRC surveillance	10	4
Other (abnormal imaging, diverticular disease, and anal prolapse)	10	4
Polyposis syndrome	7	3
EGD	96	20
UGI symptoms without warning signs (nausea/vomiting, abdominal pain, dyspepsia, and GERD)	22	23
Variceal screening or surveillance	21	22
UGI symptoms with warning signs (dysphagia, odynophagia, obstruction, and weight loss)	13	14
Other (follow-up intestinal metaplasia, *Helicobacter pylori* follow-up, and polyposis syndrome)	12	13
Barrett's esophagus screening or surveillance	8	8
Gastric ulcer follow-up and gastric cancer screening	8	8
Bleeding (melena, hematemesis, and IDA)	7	7
Abnormal imaging	5	5
Colonoscopy and EGD	106	22
Bleeding (hematemesis, melena, BRBPR, and IDA)	32	30
UGI symptoms without warning signs (nausea/vomiting, abdominal pain, dyspepsia, and GERD)	20	19
Other (abnormal imaging, variceal screening/surveillance, CRC screening/surveillance, and weight loss)	21	20
LGI symptoms (changes in stool, abdominal pain, and chronic diarrhea)	13	11
Inflammatory bowel diseases (Crohn's and ulcerative colitis)	7	7
Polyposis syndrome	7	7
UGI symptoms with warning signs (dysphagia, odynophagia, and obstruction)	6	6
Flex Sig/EUS/ERCP/SBE/DBE/VCE	44	9
Abnormal imaging	17	38
Other (CRC screening/surveillance, polyposis syndrome, and abnormal FIT)	13	29
Inflammatory bowel diseases (Crohn's and ulcerative colitis)	8	18
Bleeding (hematemesis and IDA)	4	9
Biliary stent exchange	3	7

BRBPR, bright red blood per rectum; CRC, colorectal cancer; DBE, double-balloon enteroscopy; EGD, esophagogastroduodenoscopy; ERCP, endoscopic retrograde cholangiopancreatography; EUS, endoscopic ultrasound; FIT, fecal immunochemical test; Flex Sig, flexible sigmoidoscopy; GERD, gastroesophageal reflux disease; IDA, iron deficiency anemia; LGI, lower gastrointestinal; SBE, single-balloon enteroscopy; UGI, upper gastrointestinal; VCE, video capsule endoscopy.

To date, 46% (223/480) of initially delayed cases have been completed. Univariate logistic regression revealed that after COVID-19-related delay, patients 65 years and older were more likely to complete an endoscopy compared with those under age 50 years (57.1% vs 42.8%, OR 1.79, CI 1.10–2.91, *P* = 0.02) and Asian patients were more likely to complete an endoscopy than white patients (62% vs 45%, OR 1.98, CI 1.05–3.72, *P* = 0.04). There were no statistical differences in endoscopy completion by sex, other races, ethnicity, primary language, procedure type, or planned sedation (see Supplemental Table 1, Supplementary Digital Content 1, http://links.lww.com/CTG/A625). Multivariate logistic regression adjusting for age, sex, race, ethnicity, primary language, procedure type, and sedation type found no statistically significant predictors of endoscopy completion (Table 2). The median time to endoscopy completion after initial delay was 88 days (IQR 63–119) without differences in time to endoscopy by procedure type (Table 3).

**Table 2.** Characteristics of patients with and without an endoscopic procedure after coronavirus disease 2019 delay

	Not Completed N = 257 (54%)	Completed N = 223 (46%)	aOR^a^	95% CI	*P* value
Gender, n (%)					
Male	130 (49.6)	132 (50.4)	Ref.		
Female	127(58.3)	91 (41.7)	0.74	0.51–1.07	0.11
Age, n (%)					
<50	79 (57.2)	59 (42.8)	Ref.		
50–64	124 (57.4	92 (42.6)	0.98	0.61–1.55	0.92
>65	54 (42.9)	72 (57.1)	1.68	1.00–2.81	0.05
Race, n (%)					
White	157 (55.1)	128 (44.9)	Ref.		
Black	40 (55.6)	32 (44.4)	0.99	0.58–1.70	0.98
Asian	18 (38.3)	29 (61.7)	1.92	0.96–3.84	0.07
AI/AN	8 (50)	8 (50)	1.22	0.43–3.45	0.71
Pacific Islander	6 (85.7)	1 (14.3)	0.21	0.02–1.80	0.15
Not available	28 (52.8)	25 (47.2)	0.70	0.26–1.92	0.49
Ethnicity, n (%)					
Non-Hispanic	208 (54.6)	173 (45.4)	Ref.		
Hispanic	26 (48.1)	28 (51.9)	1.53	0.74–3.15	0.25
Not available	23 (51.1)	22 (48.9)	1.63	0.56–4.73	0.37
Language, n (%)					
English	222 (54.8)	183 (45.2)	Ref.		
Non-English	35 (46.7)	40 (53.3)	1.11	0.60–2.06	0.75
Procedure type, n (%)					
Colonoscopy	118 (50.4)	116 (49.6)	Ref.		
EGD	53 (55.2)	43 (44.8)	0.74	0.43–1.25	0.26
Colonoscopy and EGD	64 (60.4)	42 (39.6)	0.68	0.041–1.12	0.13
Other	22 (50)	22 (50)	1.11	0.53–2.34	0.77
Planned sedation, n (%)					
Moderate sedation	128 (50.8)	124 (49.2)	Ref.		
Anesthesia	126 (56.5)	97 (43.5)	0.95	0.63–1.44	0.82
None	3 (60.0)	2 (40.0)	0.64	0.09–4.68	0.66

AI/AN, American Indian/Alaska Native; aOR, adjusted odds ratio; CI, confidence interval; EGD, esophagogastroduodenoscopy.

a Adjusted for sex, age, race, ethnicity, language, procedure type, and planned sedation.

**Table 3.** Median time to procedure completion after initial delay by procedure type

Procedure Type	Total (N)	Median (IQR)	*P* value
Colonoscopy	116	91 (67–119)	Ref.
EGD	43	83 (57–112)	0.12
EGD and colonoscopy	42	91 (66–122)	0.93
Other	22	96 (48–114)	0.64
Total	223	88 (63–119)	

EGD, esophagogastroduodenoscopy; IQR, interquartile range.

Of the patients who have completed an endoscopic procedure, 5.4% (12/223) were diagnosed with colorectal (n = 9), pancreatic (n = 2), and stomach (n = 1) cancers. No patients were diagnosed with active inflammatory bowel disease on endoscopy or pathology. Of the patients who have not completed an endoscopy, 11% (28/257) presented to the emergency department at a median of 149 days (IQR 49–205) after procedure delay and 39% (11/28) were for GI reasons (diarrhea, abdominal pain, and incontinence). The most frequent indications for initial endoscopies in this population were colorectal cancer screening or polyp surveillance (n = 7), iron deficiency anemia (n = 4), and diarrhea (n = 3). In addition, 8% (20/257) were admitted to the hospital at a median 154 days (IQR 50–205) after procedure delay and 15% (3/20) were admitted for GI symptoms including 1 patient who was diagnosed with rectal cancer during their hospitalization. The most frequent indications for initial endoscopies in this population were colorectal cancer screening or polyp surveillance (n = 10) and inflammatory bowel disease surveillance (n = 3).

## DISCUSSION

In this retrospective analysis of real-world data collected throughout the initial surge of the COVID-19 pandemic, we report 480 patients with procedure delays in an urban tertiary, healthcare system. These procedures were scheduled for colorectal cancer screening, suspected GI bleeding, inflammatory bowel disease management, and more. To date, 46% of patients have completed their endoscopy, and 12 patients have been diagnosed with new GI cancers. We found no statistically significant differences in endoscopy completion by sociodemographic factors, procedure type, or sedation type. We also found no statistical differences in time to repeat endoscopy by procedure type. A minority of patients presented to the emergency department were hospitalized after endoscopic procedure delays, primarily because of non-GI signs or symptoms.

To minimize potential losses to follow-up, delayed, or missed cancer diagnoses and to reduce progression of GI diseases, it is essential that endoscopic procedures delayed during the pandemic are closely tracked and rescheduled because local COVID-19 regulations allow. A survey of GI practices in the United States found variable institutional responses to COVID-19, and two-thirds of respondents had no defined plan to address the postponement of nonurgent endoscopy procedures (11). In our healthcare system, the response to the impending procedural backlog created by COVID-19 was to prospectively track delayed cases, triage by acuity of procedure indication, and to reschedule patients as soon as regulations allowed. To date, almost half of initially delayed cases have been completed with no significant differences by sociodemographic factors, and staff continue to prioritize rescheduling the remaining patients.

Tailored efforts including multipronged approaches may help reduce the potential public health impact of delayed endoscopic procedures for GI diseases (12). Although healthcare systems work to reschedule delayed endoscopic procedures, institutions can further address delays by increasing the use of noninvasive colorectal cancer screening tests, increasing gastroenterology clinical and administrative staffing, offering evening or weekend endoscopy sessions (13), and framing healthcare system messages to address patient fear (14). These proactive approaches are necessary not only to help minimize endoscopy wait times for patients at risk of worsening GI disease but are critical in averting other public health crises from preventable diseases and exacerbating racial, ethnic, and socioeconomic disparities in GI diseases (15).

The strengths of this study include a comprehensive evaluation of the impact of COVID-19 on endoscopic delays in an urban, safety-net county teaching hospital and a cancer center both affiliated with a tertiary academic center. Our data were enriched by a centralized EHR, a homegrown database to track delayed procedures, and pathology reports for patients who completed endoscopic procedures. There are several limitations worth noting. First, the unique patient settings may limit the generalizability of our findings. Second, out-of-network utilization, especially in patients over 65 years who qualify for Medicare, may not have been completely captured in this study. Third, the number of delayed procedures reported in our study was modest. This is likely due to ongoing procedures defined as urgent or emergent by the professional gastroenterology associations during our study period. Despite these limitations, we believe these findings are valuable and can inform quality improvement efforts for gastroenterology practices.

In this retrospective cohort study, colonoscopies for colorectal cancer screening, EGDs for upper GI symptoms, and combined colonoscopy and EGD for suspected bleeding were the most commonly delayed endoscopic procedures at the peak of the COVID-19 pandemic. In our cohort, 46% of patients with delayed cases have completed an endoscopic procedure to date with 12 colorectal, pancreatic, and stomach cancers diagnosed, highlighting the real-world impact of pandemic-related procedural delays. To minimize delayed or missed diagnoses and to reduce the progression of GI diseases, all efforts should be used to minimize loss to follow-up in those whose endoscopic procedures were delayed because of COVID-19.

## CONFLICTS OF INTEREST

**Guarantor of the article:** Rachel B. Issaka, MD, MAS.

**Specific author contributions:** Study concept and design: R.B.I., L.D.F., G.K., Y.T., and L.S. Acquisition, analysis, and interpretation of data: R.B.I., L.D.F., J.K., E.H., B.S., and G.K. Drafting of the article: R.B.I. and L.D.F. Critical revision of the article for important intellectual content: All authors. Approval of the final article: All authors.

**Financial support:** R.B.I. receives funding from National Institutes of Health/National Cancer Institute award number K08 CA241296. Role of the Funder/Sponsor: The content is solely the responsibility of the authors and does not necessarily represent the official views of the National Institutes of Health. The funder had no role in the design and conduct of the study; collection, management, analysis, and interpretation of the data; preparation, review, or approval of the article; and decision to submit the article for publication.

**Potential competing interests:** None reported.

**Ethics approval:** This study was reviewed and approved by our Institutional Review Board. As the study was deemed to be of minimal risk, a waiver of written informed consent was granted.

**Study Highlights**

**WHAT IS KNOWN**

✓ Coronavirus disease 2019 (COVID-19) has led to significant declines in endoscopic procedures.
✓ These declines could lead to more advanced stage cancer diagnoses and progression of gastrointestinal diseases.

**WHAT IS NEW HERE**

✓ In a tertiary academic institution, 480 nonurgent endoscopic procedures were delayed at the peak of the COVID-19 pandemic.
✓ To date, 46% of delayed cases have been completed with 12 gastrointestinal cancers diagnosed, and no differences in endoscopic completion by sociodemographic factors, procedure type, or planned sedation.

**TRANSLATIONAL IMPACT**

✓ Proactive measures throughout the pandemic could enable gastroenterology practices to determine the impact of COVID-19 on clinical practice.
✓ Tailored efforts may be required to ensure patients with COVID-19-related endoscopic delays are not permanently lost to follow-up.

## Supplementary Material

SUPPLEMENTARY MATERIAL
