# Supplementary material for: Real-World Data on the Impact of COVID-19 on Endoscopic Procedural Delays
Source: Clin Transl Gastroenterol. 2021 Jun 1;12(6):e00365. doi: 10.14309/ctg.0000000000000365 (PMC8162484; doi:10.14309/ctg.0000000000000365)
Supplement: SUPPLEMENTARY MATERIAL [file ct9-12-e00365-s001.docx]

**Supplemental Table 1:** Procedures completed during the initial COVID-19 delay

| **Procedure Type** | Total (N=268) | |
| --- | --- | --- |
|  | **n** | **%** |
| Colonoscopy | 59 | 22% |
| EGD | 108 | 40% |
| Colonoscopy & EGD | 35 | 13% |
| Other | 66 | 25% |
| *Flex Sig* | 41 |  |
| *ERCP/EUS* | 20 |  |
| *SBE/DBE/VCE* | 5 |  |

*EGD = esophagogastroduodenoscopy; Flex Sig = flexible sigmoidoscopy; EUS = endoscopic ultrasound; ERCP = endoscopic retrograde cholangio-pancreatography; SBE = single balloon enteroscopy; DBE = double balloon enteroscopy; VCE = video capsule endoscopy*

**Supplemental Table 2:** Characteristics of patients with delayed procedures

|  | **Overall (%)**  **N=480** | **Not Completed**  **N=257 (54%)** | **Completed**  **N=223 (46%)** | **OR** | **95%CI** | **p-value** |
| --- | --- | --- | --- | --- | --- | --- |
| **Gender, n (%)** |  |  |  |  |  |  |
| Male | 262 (55%) | 130 (49.6%) | 132 (50.4%) | Ref. |  |  |
| Female | 218 (45%) | 127(58.3%) | 91 (41.7%) | 0.71 | 0.49-1.01 | 0.06 |
| **Age, n (%)** |  |  |  |  |  |  |
| <50 | 138 (29%) | 79 (57.2%) | 59 (42.8%) | Ref. |  |  |
| 50-64 | 216 (45%) | 124 (57.4%) | 92 (42.6%) | 0.99 | 0.65-1.53 | 0.98 |
| >65 | 126 (26%) | 54 (42.9%) | 72 (57.1%) | 1.79 | 1.10-2.91 | 0.02 |
| **Rac**e, **n (%)** |  |  |  |  |  |  |
| White | 285 (59%) | 157 (55.1%) | 128 (44.9%) | Ref. |  |  |
| Black | 72 (15%) | 40 (55.6%) | 32 (44.4%) | 0.99 | 0.58-1.65 | 0.94 |
| Asian | 47 (10%) | 18 (38.3%) | 29 (61.7%) | 1.98 | 1.05-3.72 | 0.04 |
| AI/AN | 16 (3%) | 8 (50%) | 8 (50%) | 1.22 | 0.45-3.36 | 0.69 |
| Pacific Islander | 7 (2%) | 6 (85.7%) | 1 (14.3%) | 0.20 | 0.02-1.72 | 0.14 |
| Not Available | 53 (11%) | 28 (52.8%) | 25 (47.2%) | 1.10 | 0.61-1.97 | 0.76 |
| **Ethnicity, n (%)** |  |  |  |  |  |  |
| Non-Hispanic | 54 (11%) | 208 (54.6%) | 173 (45.4%) | Ref. |  |  |
| Hispanic | 381 (79%) | 26 (48.1%) | 28 (51.9%) | 1.29 | 0.73-2.29 | 0.38 |
| Not Available | 45 (10%) | 23 (51.1%) | 22 (48.9%) | 1.15 | 0.62-2.13 | 0.66 |
| **Language, n (%)** |  |  |  |  |  |  |
| English | 405 (84%) | 222 (54.8%) | 183 (45.2%) | Ref. |  |  |
| Non-English | 75 (16%) | 35 (46.7%) | 40 (53.3%) | 1.39 | 0.85-2.27 | 0.20 |
| **Procedure Type, n (%)** |  |  |  |  |  |  |
| Colonoscopy | 234 (49%) | 118 (50.4%) | 116 (49.6%) | Ref. |  |  |
| EGD | 96 (20%) | 53 (55.2%) | 43 (44.8%) | 0.83 | 0.51-1.33 | 0.43 |
| Colonoscopy & EGD | 106 (22%) | 64 (60.4%) | 42 (39.6%) | 0.67 | 0.42-1.06 | 0.09 |
| Other | 44 (9%) | 22 (50%) | 22 (50%) | 1.02 | 0.53-1.94 | 0.96 |
| **Planned Sedation, n (%)** |  |  |  |  |  |  |
| Moderate Sedation | 252 (53%) | 128 (50.8%) | 124 (49.2%) | Ref. |  |  |
| Anesthesia | 223 (46%) | 126 (56.5%) | 97 (43.5%) | 0.79 | 0.55-1.14 | 0.21 |
| None | 5 (1%) | 3 (60.0%) | 2 (40.0%) | 0.69 | 0.11-4.19 | 0.69 |

EGD = esophagogastroduodenoscopy
